# The Mediterranean Diet Slows Down the Progression of Aging and Helps to Prevent the Onset of Frailty: A Narrative Review

**DOI:** 10.3390/nu12010035

**Published:** 2019-12-21

**Authors:** Cristiano Capurso, Francesco Bellanti, Aurelio Lo Buglio, Gianluigi Vendemiale

**Affiliations:** Department of Medical and Surgical Sciences, University of Foggia, Viale Pinto 1, 71122 Foggia, Italy; francesco.bellanti@unifg.it (F.B.); aurelio.lobuglio@unifg.it (A.L.B.); gianluigi.vendemiale@unifg.it (G.V.)

**Keywords:** Mediterranean diet, aging, frailty, sarcopenia

## Abstract

The aging population is rapidly increasing all over the world. This results in significant implications for the planning and provision of health and social care. Aging is physiologically characterized by a decrease in lean mass, bone mineral density and, to a lesser extent, fat mass. The onset of sarcopenia leads to weakness and a further decrease in physical activity. An insufficient protein intake, which we often observe in patients of advanced age, certainly accelerates the progression of sarcopenia. In addition, many other factors (e.g., insulin resistance, impaired protein digestion and absorption of amino acids) reduce the stimulation of muscle protein synthesis in the elderly, even if the protein intake is adequate. Inadequate intake of foods can also cause micronutrient deficiencies that contribute to the development of frailty. We know that a healthy eating style in middle age predisposes to so-called “healthy and successful” aging, which is the condition of the absence of serious chronic diseases or of an important decline in cognitive or physical functions, or mental health. The Mediterranean diet is recognized to be a “healthy food” dietary pattern; high adherence to this dietary pattern is associated with a lower incidence of chronic diseases and lower physical impairment in old age. The aim of our review was to analyze observational studies (cohort and case–control studies) that investigated the effects of following a healthy diet, and especially the effect of adherence to a Mediterranean diet (MD), on the progression of aging and on onset of frailty.

## 1. Introduction

Population aging is now a global phenomenon that is rapidly evolving all over the world. In the European Union, the number of people over sixty-five is expected to increase from 85 million in 2008 to 151 million in 2060 [1]. Worldwide, people aged over 65 are estimated to increase from 461 million in 2004 to 2 billion by 2050 [2,3]. This will have important consequences for the planning and delivery of health care and social assistance services. A greater life expectancy, in fact, leads us to reconsider not only the condition of the elderly, but also what kind of implications aging will have in our lifetime [4,5]. That is, the ability to dedicate ourselves to our activities will be no different from that of a younger person, wherever we spend our old age in good health. On the contrary, where the years of old age are dominated by loss of health, cognitive decline and therefore, the loss of self-sufficiency, the implications for individuals and for society as a whole would be much more negative [5]. Especially in high-income countries, it is now customary for many people to spend their old age in innovative ways, such as starting a new career or even starting new studies, or devoting oneself completely to a neglected passion during working age [6]. It is clear, however, that the extent of these new opportunities arising from these long years of life depends, for everyone, on maintaining health and self-sufficiency.

Healthy behaviors in middle age predispose to so-called “healthy and successful” aging, which is the condition of the absence of serious chronic diseases or of important decline in cognitive or physical functions, or mental health [7]. Healthy behaviors include following a healthy diet, with an adequate caloric restriction to the state of health and physical activity, not smoking, intake of moderate amounts of alcohol, especially in women. Akbaraly et al. [8], and Samieri et al. [9] from the results of the Whitehall II cohort study and of the Nurses’ Health Study, respectively, showed that a healthy dietary pattern, like the Mediterranean Diet (MD) pattern, was associated to a lower incidence of chronic disease and to a lower physical impairment in old age. In addition, they showed that a healthy dietary pattern was associated to a lower cardiovascular risk and to a lower risk of premature death.

The aim of our narrative review was to analyze observational studies (cohort and case-control studies) that investigated the effects of following a healthy diet, and especially the effect of adherence to MD, on the progression of aging and on the onset of frailty.

## 2. Aging and Frailty

**Figure 1:**
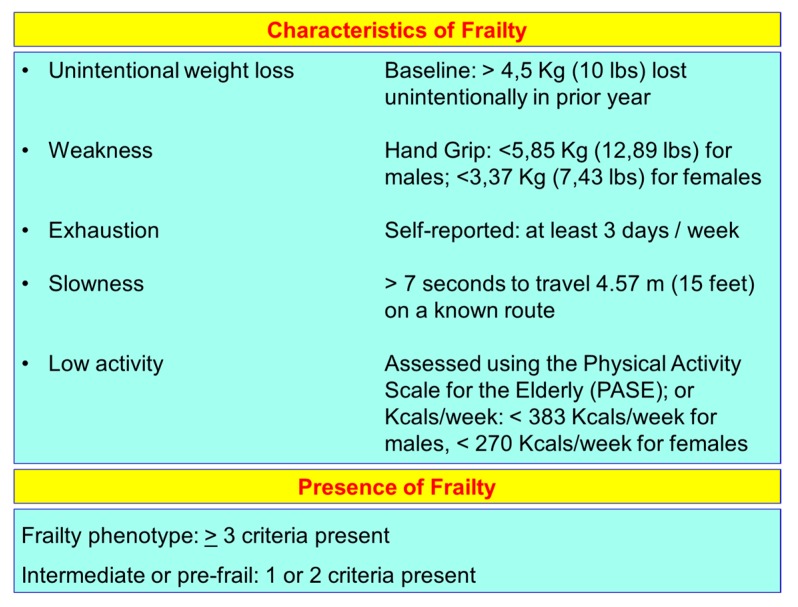
Fried’s criteria of frailty (line 69).

**Figure 2:**
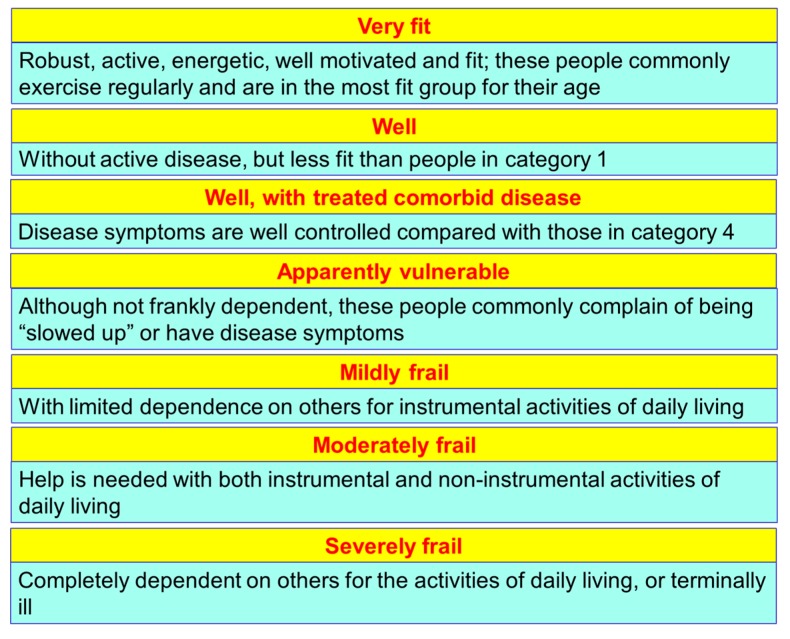
The Canadian Study of Health and Aging (CSHA) Clinical Frailty Scale by Rockwood.

The frailty of the elderly is a condition characterized by an increased vulnerability to poor homeostasis resolution after a stress event, which increases the risk of negative outcomes, including falls, delirium and disability. Most geriatricians intuitively recognize frailty; otherwise, this is mostly misunderstood, or confused with the presence of comorbidity and disability [10,11,12]. With the aim to provide a standardized definition for frailty, Fried et al. [13] identified the “frailty phenotype”, which is the measure most frequently used. Fried’s criteria of frailty considers five items to determine the level of frailty: weight loss, exhaustion, low physical activity, slowness, and weakness (Figure 1). Rockwood et al. [14] starting from the identification, through the Canadian Study on Health and Aging (CSHA), of 70 items including signs, symptoms and abnormal tests that characterized the fragility, built their “CSHA Clinical Frailty Scale”, which considered seven levels, form Very fit to Severely frail (Figure 2). More correctly, frailty develops due to the greater decline, already linked to age, up to the severe impairment and the appearance of pathological states, of the different organs and systems, which overall, leads to a condition of greater vulnerability and reduced resilience and ultimately, to sudden and severe health changes triggered by even mild stressors. In the frail elderly an apparently small insult, for example, a small infection or minor surgery produces a significant worsening of the patient’s condition. This means that the frail elderly loses all self-sufficiency, undergoes a hypokinetic syndrome to the condition of entrapment, increases the risk of falls, and develops a state of confusion, with a serious deterioration of cognitive functions. [15]. Frail people have several functional deficits, which often cause falls, immobility and confusion. They are the patients who come to the hospital with greater frequency and are the main recipients of long-term care services. [16]. About 25–50% of people over the age of 85 are estimated to have different degrees of frailty; these are subjects at high risk of falling, permanent disability and death, in most cases requiring long-term care [13,15]. However, if we consider that 25–50% of people over the age of 85 are frail, we must consider that that 50% to 75% of people over 85 years are not frail, which raises several questions, namely: How does frailty develop? How we can prevent it? How can we reliably detect it?

**Figure 3:**
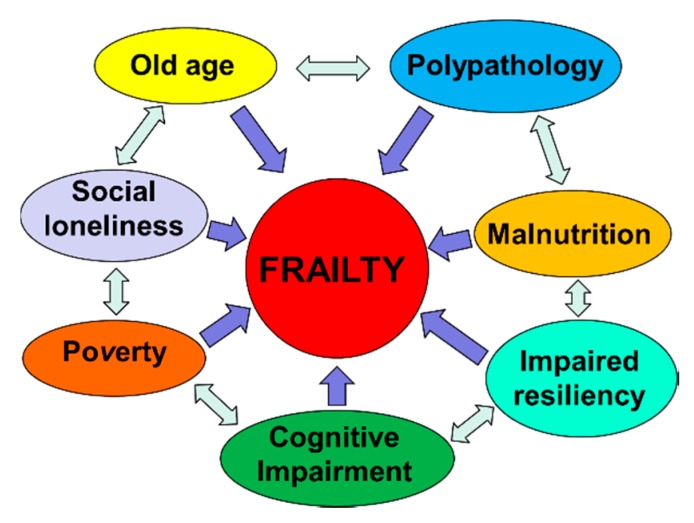
The vicious circle of frailty (line 92).

Frailty must not be understood as an inevitable consequence of aging, but rather, must be understood as a geriatric syndrome [13,17,18,19]. It is therefore more appropriate to speak of “frailty syndrome”, i.e., a chronic pathological condition that results from the interaction of various factors, including aging-related physiological alterations, poly-pathology, nutritional deficiencies up to severe malnutrition, and the negative impact of socio-environmental factors (Figure 3).

## 3. Aging, or Cellular Senescence, and Health

If we consider aging solely from the biological point of view, we refer essentially to the physiological and progressive accumulation of senescent cells in tissues and organs, which occurs during the life of each individual [19], with the progressive slowing down and, in some cases, loss of function. The antagonist pleiotropic genes are a set of genes that regulate cellular senescence [20,21,22,23]; these genes play an important role in the prevention of malignant cell degeneration in the pre-neoplastic degeneration, i.e., eliminating cells from the cell cycle; the same genes are also involved in the mechanisms of protection in the physiological cellular senescence processes and towards age-related diseases [24,25,26].

The reduction of the capacity of cell regeneration and tissue repair are the basis of the physiological senescence process, substantially due to the slowdown process, up to the complete stop of the cycle of growth and replication of the progenitor cells. These aging cells also produce a series of proinflammatory and lytic molecules of the extracellular matrix in the process known as the secretory phenotype associated to senescence (SASP). Consequently, those mechanisms normally needed to maintain tissue homeostasis in the aging organism produce a series of alterations in the structure and function of the cells, resulting in degeneration and pathological senescence. The aging process also involves the immune system; in particular, the cell-mediated defense mechanism is slowed down. Furthermore, senescent cells do not produce enough signals to activate immune cells. As a result, a few aging senescent cells survive in the body. The senescent cells, in addition to reducing the functional efficiency of the organs and systems during aging, make them more vulnerable, therefore subject to further deterioration, after exposure to environmental stress factors [27].

In physiological aging, senescence is induced by the accumulation of different degenerative factors that slowly accumulate in the cells and that are responsible for macromolecular damage [28,29,30], for example, the shortening of telomeres, the addition of secondary DNA alterations to oxidative damage, stress degeneration of the endoplasmic reticulum (ER). The disease takes place when environmental stressors attack the tissues already in the presence of senescent cells with very low resilience capacities [31,32,33]. The stress that can cause the disease can be foreign and abnormal to the cell, as damaging DNA agents in cigarette smoke; otherwise, it can be a more intense response to a stressor event prolonged over time by the same factors that work in physiological aging, e.g., erosion of telomeres in lung epithelial cells chronically damaged by smoke [34]. It is therefore evident that aging is the result of a multi-factorial interaction between local and systemic environmental factors, and involutional factors due to cell senescence. Over time, these environmental stressors add to the cellular changes linked to physiological aging, decreasing resistance to stress and further reducing tissue resilience. That is, the chronic stimulation of a stressor factor on a tissue already in a condition of cellular senescence is more likely to induce the onset of a pathological condition [27].

## 4. The Role of Senescence in the Progression of Diabetes Mellitus and Atherosclerosis

Under physiological conditions, the synthesis and release of insulin by pancreatic beta cells, based on glycemic levels, allows the absorption of glucose in sensitive peripheral tissues, i.e., the liver and skeletal muscle, with the consequent production of energy by means of aerobic glycolysis [35]. In obese people, build-up of abdominal fat tissue, which is characterized by a chronic inflammatory state, and the excess of free circulating fatty acids, can cause a state of peripheral tissue insulin resistance [36,37]. Thus, what occurs is a condition of increased and abnormal prolonged demand in the time of production and release of insulin with the consequent prolonged stimulation of pancreatic beta cells. The increased replication activity, and consequently, the wear of telomeres, leads to premature senescence of beta cells [38], thus reducing the efficacy of the reaction to hyperglycaemia with the consequent aggravation of type 2 diabetes mellitus (T2DM) and the onset of insulin resistance. This reduced ability to regulate blood glucose levels alters the metabolic processes of the cells and speeds up the aging process. This glucose-induced cellular toxicity induces a condition of chronic stress and consequently, pathological senescence in various cell lines, for example, fibroblasts, cells of the renal tubules, endothelial cells, mesenchymal cells [39,40,41,42], and even neurons. Therefore, we see the appearance and progression of a number of diseases, such as atherosclerosis, chronic renal failure [43] and Alzheimer’s disease. Furthermore, the accumulation of abdominal adipose tissue and the release in circulation of excess free fatty acids can also cause the progression of atherosclerosis. During the formation and progression of plaque, the proliferation of smooth muscles and the reduction of endothelial nitric oxide synthase levels can lead to the shortening of telomeres and increased oxidative stress, respectively [44,45]. Plaque progression is therefore the result of a series of interactions between smooth muscle cells and immune system cells, modulated by the synthesis and release of cytokines and adhesion molecules by endothelial cells, smooth muscle cells and immune cells. The complexity of the action mechanisms of senescent cells in the atherogenesis process is evident. Paradoxically, senescence in atherosclerosis acts as an initial protective factor by restricting and slowing the development of plaques and minimizing plaque-disrupting apoptosis. After a limit threshold of senescence burden, the proinflammatory matrix-degrading SASP exacerbates disease [27].

## 5. Caloric Restriction, Effects on Metabolism of Adipose Tissue and Increase of Longevity

It is known that overweight at young and middle age, by decreasing insulin sensitivity, can accelerate the aging process and predispose to the onset of age-related diseases [45,46,47,48]. In experimental models conducted both in mice and in humans, it has been observed that caloric restriction (CR) produces significant effects in terms of weight reduction; much of the weight loss comes from the reduction of white adipose tissue (WAT) and visceral fat deposits [45,49,50]. It is true that the common strains of laboratory rodents all have an extended lifespan in response to CR, however, this is not a universal effect. Interestingly, some inbred mice strains show only a modest effect on extension of the life span after CR. In some cases, a reduction in life span has even been observed [51]. We can explain these apparently conflicting results considering that the effect of CR on the metabolism is a decrease in carbohydrate dependence and a greater use of fatty acid oxidation to produce energy [52]. Indeed, the level of fat reduction following dietary restriction remains a key component of the beneficial effects of CR. In a study conducted on 40 strains of mice, increased longevity induced by CR was more evident in old mice (15–17 months of age) that, with a 40% CR diet, were able to retain more fat deposits, showing that retainment of both fat mass and lean mass in aging can correlate with more longevity [53]. Subsequently, the results of a study conducted on progeroid mice showed that a caloric restriction regimen of 30% increased its median duration and the maximum residual life expectancy by three times. Specifically, mice subjected to caloric restriction retained 50% more neurons and maintained full motor function, far beyond the lifespan of ad libitum-fed mice.

## 6. Caloric Restriction and Inflammatory State

Gene expression analysis has shown that dietary restriction increases DNA resistance to stress-induced damage, as well as improving antioxidant defenses and metabolic processes primarily mediated by insulin, as well as by other hormonal signaling pathways. Dietary restriction also affects mitochondria activity, modulates apoptotic response and modifies pro-inflammatory and anti-inflammatory cytokine production [54]. The results of further randomized human studies confirm that caloric restriction stimulates the body to adapt to the use of any available energy substrate, both glucose and fatty acids [55]. The term “metabolic flexibility” optimally defines this adaptation property, which has long been linked to metabolic health and longevity [56]. Studies conducted on primates have also shown that a strong relationship exists between CR and the reduction of the pro-inflammatory state [57]. A subsequent study linked the reduction of the pro-inflammatory state to the inhibition of the inflammasome, induced, in turn, by the increase in serum levels of β-hydroxybutyrate during fasting [58]. Another suggested a mechanism through which CR could suppress the harmful activation of the immune system, which concerns the preservation of the thyme function induced by the CR, thus preserving the function of T cells [59], or a reduction of inflammation induced by oxidative stress [60].

## 7. Caloric Restriction, Mitochondria Activity and Reactive Oxygen Species Production

Numerous experimental models conducted on animal organisms have involved mitochondrial proteins encoded by the nucleus in the life span adjustment process [61,62]. These experiments on genes mostly involved an alteration of the electron transport chain process with consequent impairment of mitochondrial function; nevertheless, they have led to a greater life expectancy [48]. Conversely, in mice models bearing significant mitochondrial genome mutations designed to significantly impair mitochondrial function, a phenotype characterized by accelerated aging was observed [63,64]. The seemingly contradictory results observed in these experimental models may be due to the variability of the alteration of mitochondrial function that was obtained from time to time. Experiments conducted on C. elegans have shown an increase in life expectancy as a result of a modest reduction of the various components of the mitochondrial electron transport, while a significant reduction, i.e., a sign of high mitochondrial damage, was associated with a reduction in the lifespan [65]. Evidence from mammalian models has shown that a slight reduction in mitochondrial function is associated with prolonging lifespan. This assumption seems to contradict the hypothesis that reactive oxygen species (ROS) support aging [66]. In fact, although mitochondrial ROS can be dangerous for life [67], it is equally clear that a slight stimulation of mitochondrial ROS production can stimulate the activation of the anti-redox mechanism with protective effects in the long term.

## 8. Caloric Restriction, Hormesis and Mitochondria Activity during Aging

“Hormesis”, from the Greek verb “ormao” that means “to stimulate”, is described as the adaptive function of a cell or an organism characterized by a biphasic dose-dependent response, following exposure to a variety of stimuli, such as toxins or other stressors. The hormetic responses, generally, show a modest stimulation of the response of the organism to low doses of a stressor, or an inhibition of the same response to high doses [68]. “Mitochesis” is defined as the hermetic response of the mitochondria, i.e., that the activation of a low level of stress can protect against major and subsequent stress [69,70,71]. It is therefore clear that ROS levels are not the only factor responsible for mitochondrial stress affecting life span. Another mechanism linking the aging of mitochondria is constituted by the turnover capacity, that is, the balance between biogenesis, or the synthesis of new mitochondria, and mitophagy, or the removal of aged and damaged mitochondria. It is now clear that all interventions, such as CR, which result in greater longevity, act by inducing mitochondrial biogenesis through the expression of peroxisome proliferation-activated receptor gamma (PPAR-γ), and coactivator 1 alpha (PGC-1α) [72,73]. It is equally clear that the reduction of biogenesis contributes to the onset of age-related diseases, both through the reduced activity of PGC-1αand through other routes. [74]. A gradual reduction in autophagic activity associated with aging was also observed [75]; a similar age-related decline is also likely to affect the more specialized process of mitophagy. On the contrary, in experimental models, the increase in the expression of gene products that stimulate mitochondrial activity has been observed to determine a prolongation of life expectancy [76].

## 9. Caloric Restriction and DNA Methylation

In addition, DNA methylation (methylation drift) is an important epigenetic modification that involves the addition of a methyl group in the cytosine 5 position by DNA methyltransferase to form 5-methylcyctosine (5-mC). This epigenetic mark has the power to activate or deactivate genes and can be inherited through cell division. DNA methylation plays an important role in normal human development, aging, tumorigenesis and other genetic and epigenetic diseases [48,77,78]. Maegawa et al. [79] found that DNA methylation drifts with age in both mice and humans and monkeys. They also found that Methylation drift is inversely proportional to lifespan across these three-mammalian species. In their elegant study, authors also found that a sample of 22 to 30-year-old rhesus monkeys exposed to 30% CR since 7–14 years of age showed attenuation of age-related methylation drift compared to ad libitum-fed controls, such that their blood methylation age appeared 7 years younger than their chronologic age. They observed the same and more pronounced effects in 2.7–3.2-year-old mice exposed to 40% CR starting at 0.3 years of age.

## 10. Caloric Restriction, Metabolic Adaptation and Oxidative Damage

Readman et al., in the CALERIE Study [80]., delivered a highly controlled and intensive behavioral trial targeting a 25% CR diet over 2 years in a sample of 53 voluntary and non-obese adults (34 CR and 19 controls); then, they measured, in a room calorimeter, the component of daily sedentary energy expenditure, i.e., the energy metabolism during sleep. They also measured hormonal mediators of metabolism, including leptin and thyroid hormones, along with urinary F2-isoprostane excretion as an index of oxidative damage. After two years, subjects in the CR group experienced an average weight loss of 8.7 Kg, whereas subjects in the control group maintained weight during the 2-year period. In the CR group, the greater weight loss was from fat mass after two years (−3.2 Kg; *p* > 0.001). In addition, the authors found a significative reduction of 24-h Energy Expenditure (−186 Kcal/day; *p* < 0.05), and a significative reduction in Sleep Energy Expenditure (−160 Kcal/day; *p* < 0.05) in the CR group after two years, indicating a metabolic adaptation to the CR. After adjustment for changes in body composition, while Sleep Energy Expenditure was significantly decreased in the CR group after two years (*p* < 0.05), the 24-h Energy Expenditure did not decrease significantly after two years, compared to the control group (*p* > 0.55). As regards the biomarkers of Energy Metabolism and aging, authors found a significant reduction in the CR group after two years of T3 (−0.73 ng/dl; *p* < 0.05), T4 (−0.16 mcg/dl; *p* < 0.05), and leptin (−9.3 ng/dl; *p* < 0.05); in addition, they observed a reduction of fasting insulin and of nigh time core body temperature. Regarding the markers of oxidative stress, the authors found a significative reduction of the urinary excretion of four F2-isoprostane isomers after two years in the CR group (−0.49 ng/mg CR; *p* < 0.05). The authors have linked these data to the metabolic adaptation of the 24-h Energy Expenditure after the CR. The author also linked the CR with an improved mitochondrial function, with a decreased total body oxygen consumption, and therefore, with a decreased reduction of Oxidative Stress.

## 11. Mediterranean Diet, Cardiovascular Disease and Mortality

The traditional Mediterranean Diet (MD) is characterized by a high intake of foods of plant origin (fruit, vegetables, breads, other cereals, potatoes, beans, nuts, and seeds) and fresh fruit. Olive oil, namely extra-virgin olive oil, is the main source of fat. Dairy products (mainly light cheeses and yogurt), fish and poultry are consumed in medium-low quantities; particularly, fish is an excellent source of polyunsaturated fatty acids (PUFAs), particularly omega-3 fatty acids; egg consumption is limited to a maximum of four per week; red meat is consumed sporadically and in small quantities, however, no more than once a week. MD has a very low saturated fat content, which represents no more than 8–10% of the total caloric intake. Caloric intake from lipids is not more than 30% of total caloric intake. Wine is usually consumed with meals, but always in moderate doses (1–2 glasses) [81,82]. Ancel Keys first demonstrated the health benefits derived from the MD [83]; thanks to early studies of Ancel Keys, MD has been proposed as a healthy dietary pattern associated with a lower risk of developing cardiovascular and metabolic diseases. The traditional MD has been proposed as a food model to achieve or maintain optimal weight. Trichopoulou et al. [84] showed that high adherence to the MD, assessed by the Mediterranean Diet Score (MDS), was related to a significative reduction of total mortality [85].

Subsequently, the PREDIMED study [86] confirmed the finding above. The authors have shown that all subjects at high cardiovascular risk who followed an MD pattern, supplemented with monounsaturated fatty acids and antioxidants, that is, extra-virgin olive oil (EVOO), or with omega-3 poly-unsaturated fatty acids, that is nuts, had a reduced Hazard Risk (HD) of acute myocardial infarction, stroke or death from any cardiovascular event (MD with EVOO: HR = 0.70, 95% CI: 0.53–0.91, *p* = 0.009; MD with nuts: HR = 0.70, 95% CI: 0.53–0.94, *p* = 0.02). They did not observe any effect on reduction of all-cause mortality (MD with EVOO: HR = 0.81, 95% CI: 0.63–1.05, *p* = 0.11; MD with nuts: HR = 0.95, 95% CI: 0.73–1.23, *p* = 0.68).

The authors subsequently identified protocol deviations, including the enrolment of household members without randomization, assignment to a study group without randomization of some participants at one of 11 study sites, and apparent inconsistent use of randomization tables at another site. Then, they published a new revised report, which was based on analyses that do not rely exclusively on the assumption that all the participants were randomly assigned [87]. In their second report, the authors confirmed the lower risk of acute myocardial infarction, ischemic stroke, or death from all cardiovascular event among subjects who were assigned to an MD pattern respect to control subjects who were assigned to a low-fat diet.

A subsequent meta-analysis [88] analyzed the relationship between adherence to MD and mortality and incidence of each disease; 1,574,299 subjects were involved; they were followed for a time ranging from three to 18 years. The authors demonstrated a significant association between greater adherence to MD, a significant improvement in health status and a significant reduction in mortality Rate Risk (RR) (RR = 0.91, 95% CI: 0.89–0.94; *p* < 0.0001). The authors have also shown that a greater adherence to an MD pattern was associated with a significant reduction in mortality due to coronary heart disease (CHD) (RR = 0.91, 95% CI: 0.87–0.95, *p* < 0.0001) and to cancer (RR = 0.94; 95% CI: 0.92–0.96; *p* < 0.0001).

A further meta-analysis conducted by the same authors [89] further showed that a great adherence to MD was associated with an improvement in health status and quality of life and to a significant reduction in overall mortality (RR = 0.92, 95% CI: 0.90–0.94, *p* < 0.00001). Specifically, authors showed a significative reduction of mortality from coronary heart disease (RR = 0.90; 95% CI: 0.87–0.93; *p* < 0.00001) and from cancer (RR = 0.94; 95% CI: 0.92–0.96; *p* < 0.00001). A high adherence to MD was also associated with a significant reduction in the incidence of neurodegenerative diseases (RR = 0.87; 95% CI: 0.81–0.94; *p* < 0.00001), including Alzheimer’s disease.

Nevertheless, as already emphasized in an excellent editorial by Voelker [90], it would be simplistic to consider the MD as a simple semi-vegan diet rich in fibers, antioxidants and proteins of vegetable origin. We observe the benefits of the MD in its cultural context, where food is part of a lifestyle.

In 2011, the Mediterranean Diet Foundation together with the forum on Mediterranean food cultures developed a consensus document that revised the pyramid of the Mediterranean diet, including cultural and lifestyle elements [91]. The authors underlined the aspects of socialization, by writing, “The aspect of conviviality is important for the social and cultural value of the meal, beyond nutritional aspects. Cooking, sitting around the table and sharing food in company of family and friends is a social support and gives a sense of community. Make cooking an important activity taking the proper time and space. Cooking can be relaxing, fun and can be done with family, friends and the loved ones’’. At least 30 min of moderate exercise during the day “as a dietary supplement” and adequate rest at night and during the day in the form of a nap after a meal are also recommended in the consent document.

Lastly, Kromhout et al. [92], further confirmed how a food model according to the features of the MD, assessed through the Mediterranean Adequacy Index, was inversely associated with mortality due to ischemic heart disease (r = −0.91). In particular, the authors confirmed the protective role of cereals (r = −0.52), vegetables (r = −0.52) and legumes (r = −0.62), in addition to the intake of moderate amounts of alcohol in the diet (r = −0.54). The authors also confirmed the association between ischemic mortality of heart disease and dietary intake of high amounts of saturated fatty acids (r = 0.83), whole milk (r = 0.84), confectionery preparations based on simple sugars (r = 0.69), animal meats, with the exception of fish (r = 0.68), and preparations based on animal meats and simple sugars, for example, processed meats (r = 0.84). Table 1 summarizes the studies we examined.

## 12. Omega-3 Poly-Unsaturated Fatty Acids and Aging

The three types of n-3 fatty acids involved in human physiology are α-linolenic acid (ALA), which is found in plant oils, eicosapentaenoic acid (EPA), and docosahexaenoic acid (DHA), both commonly found in marine oils.

As summarized in Table 2, since 1999, the Gruppo Italiano per lo Studio della Sopravvivenza nell’Infarto Miocardico (GISSI)-Prevenzione trial, [93] an open-label trial involving 11,323 survivors of MI, reported that patients who received supplementation with omega-3 Fatty Acids (FAs) experienced a 10% reduced risk of major cardiovascular events compared with untreated controls.

The Japan EPA Lipid Intervention Study (JELIS) trial [94] is an open-label trial involving 18,645 participants with total cholesterol of 6.3 mmol/L (243.24 mg/dL) or greater, of whom only 20% had prior CHD. Authors showed that among patients who were given EPA treatment, major coronary events were reduced by 19% (EPA group vs. controls: HR = 0.81; 95% CI: 0.69–0.95; *p* = 0.011). In patients with a history of coronary artery disease who were given EPA treatment, major coronary events were reduced by 19% (EPA group vs. controls: HR = 0.81; 95% CI: 0.66–1.00; *p* = 0.048). In patients with no history of coronary artery disease, EPA treatment reduced major coronary events by 18%, but this finding did not reach statistical significance (EPA group vs. controls: 0.82 (0.63–1.06) HR = 0.82; 95% CI: 0.63–1.06; *p* = 0.132).

Seven large randomized trials [95,96,97,98,99,100,101] were conducted to compare the associations of treatment with omega-3 FAs supplementation vs. placebo or no treatment for at least 12 months in populations with prior CHD, stroke, or high risk of cardiovascular disease (CVD). These large trials all failed to demonstrate a significative reduction of the incidence of major cardiovascular events.

In addition, four meta-analyses involving large trials of omega-3 FA supplements [102,103,104,105] reported conflicting results demonstrating a significative reduction of overall mortality, of mortality due to myocardial infarction, and of sudden death in patients with coronary heart disease.

Zhang et al. conducted a large perspective cohort study involving 240,729 men and 180,580 women [106], who were prospectively followed for 16 years to examine the associations of fish and long chain omega-3 Poly-unsaturated Fatty Acids (LCn-3 PUFAs) intakes with total and cause-specific mortality.

They found that comparing the highest with the lowest quintiles of fish and LCn-3 PUFAs intake, men had lower total mortality (Fish intake Multivariable HR: 0.91, 95% CI: 0.89–0.94, *p* < 0.0001; LCn-3 PUFAs Multivariable HR: 0.89, 95% CI: 0.86–0.92, *p* < 0.0001), lower cardiovascular disease (CVD) mortality (Fish intake Multivariable HR: 0.90, 95% CI: 0.85–0.94, *p* < 0.0001; LCn-3 PUFAs Multivariable HR: 0.85, 95% CI: 0.80–0.90, *p* < 0.0001), lower cancer mortality (Fish intake Multivariable HR: 0.94, 95% CI: 0.90–0.99, *p* = 0.038; LCn-3 PUFAs Multivariable HR: 0.95, 95% CI: 0.90–1.00, *p* = 0.040), lower respiratory disease mortality (Fish intake Multivariable HR: 0.80, 95% CI: 0.72–0.89, *p* < 0.0001; LCn-3 PUFAs Multivariable HR: 0.73, 95% CI: 0.65–0.83, *p* < 0.0001), lower Alzheimer’s disease mortality (Fish intake Multivariable HR: 0.76, 95% CI: 0.61–0.95, *p* = 0.0028; LCn-3 PUFAs Multivariable HR: 0.70, 95% CI: 0.54–0.89, *p* = 0.0008), and lower chronic liver disease mortality (Fish intake Multivariable HR: 0.63, 95% CI: 0.47–0.83, *p* = 0.0013; LCn-3 PUFAs Multivariable HR: 0.66, 95% CI: 0.49–0.89, *p* = 0.0046).

Similarly, comparing the highest with the lowest quintiles of fish and LCn-3 PUFAs intake, women had lower total mortality (Fish intake Multivariable HR: 0.92, 95% CI: 0.88–0.95, *p* < 0.0001; LCn-3 PUFAs Multivariable HR: 0.90, 95% CI: 0.86–0.94, *p* < 0.0001), lower CVD mortality (Fish intake Multivariable HR: 0.90, 95% CI: 0.83–0.97, *p* = 0.0034; LCn-3 PUFAs Multivariable HR: 0.82, 95% CI: 0.75–0.90, *p* < 0.0001), lower respiratory disease mortality (Fish intake Multivariable HR: 0.81, 95% CI: 0.71–0.92, *p* < 0.0001; LCn-3 PUFAs Multivariable HR: 0.74, 95% CI: 0.64–0.87, *p* < 0.0001), and lower Alzheimer’s disease mortality (Fish intake Multivariable HR: 0.62, 95% CI: 0.48–0.80, *p* < 0.0001; LCn-3 PUFAs Multivariable HR: 0.59, 95% CI: 0.43–0.80, *p* = 0.0024). Authors explained the discrepancies with the previous studies because of the limited statistical power of those previous small-scale studies. Moreover, another cause could be partially due to different cooking methods, as fried fish which is common in North American and European countries, may produce trans-fatty acids, oxidation of PUFAs, advanced glycation products (AGEs) and increase energy density, which counteract or even reverse the beneficial effects of nutritional ingredients in fish. They found a positive correlation between fried fish intake and mortality from all causes (P-trend = 0.011), CVD (P-trend = 0.019), respiratory disease (P-trend = 0.031) and infections (P-trend = 0.020) in women. They did not find any significant relationship between intakes of fish and LCn-3 PUFAs and total mortality among participants with diabetes or BMI ≥ 30.

After examining findings from the Randomized Controlled Trials (RCT) concerning the prevention of CHD among patients at high CVD risk, coauthors of the Science Advisory from the American Heart Association [107] concluded that omega-3 PUFA supplements might reduce CHD death, by a reduction of sudden cardiac death, among patients with prior CHD. Nevertheless, the treatment does not reduce the incidence of recurrent nonfatal myocardial infarction. Because the benefit outweighs any risk of treatment, the majority of coauthors concluded that treatment with omega-3 PUFA supplements is reasonable for the secondary prevention of CHD death (Class IIa Recommendation); a minority of coauthors preferred a lower strength of recommendation for treatment of patients with this indication (Class IIb Recommendation).

In addition, coauthors examined results from the GISSI-HF RCT [108]. This RCT showed that, among patients with chronic heart failure with reduced ejection fraction, the omega-3 PUFA supplementation reduced the risk of total mortality (death resulting from any cause) by 9% (RR, 0.91; 95% CI, 0.833–0.998; *p* = 0.041) and the risk of cardiovascular-related hospitalizations or death by 8% (RR, 0.92; 95% CI, 0.849–0.999; *p* = 0.009).

They concluded that, although based on a single, large RCT, treatment with omega-3 fatty acids could be reasonable in the secondary prevention of heart failure-related hospitalizations and death among patients with heart failure with reduced ejection fraction (Class IIA, Recommendation).

Nevertheless, the coauthors recommended the need for additional RCTs among patients with heart failure and preserved ejection fraction to confirm the strength of the Recommendation.

Regarding the association between the intake of omega-3 polyunsaturated fatty acids with the reduction of the incidence of cardiovascular disease and stroke and other aging-related diseases, Qi et al. [109] showed that treatment with alpha-linolenic acid (ALA), produced a 30% increase in mean lifespan in Caenorhabditis elegans. Authors showed that ALA treatment enhanced the lifespan of the wild-type worms by activating the NHR-49/PPARα and SKN-1/Nrf2 transcription factors. Specifically, ALA activated NHR-49 to promote the expression of genes involved in the b-oxidation of lipids. ALA exposure to air also causes oxidation of ALA in a group of compounds called oxylipins. The activation of SKN-1 by oxylipins improves longevity. This study has shown that omega-3 fatty acids slow aging; this effect can be due to the association of the effects of both omega-3 fatty acids and the oxylipin metabolites. The authors suggest the hypothesis that in humans, the healthy benefits of omega-3 fatty acids intake could also derive from the production of oxylipin.

## 13. Mediterranean Diet Increases Lifespan and Improves Aging

**Figure 4:**
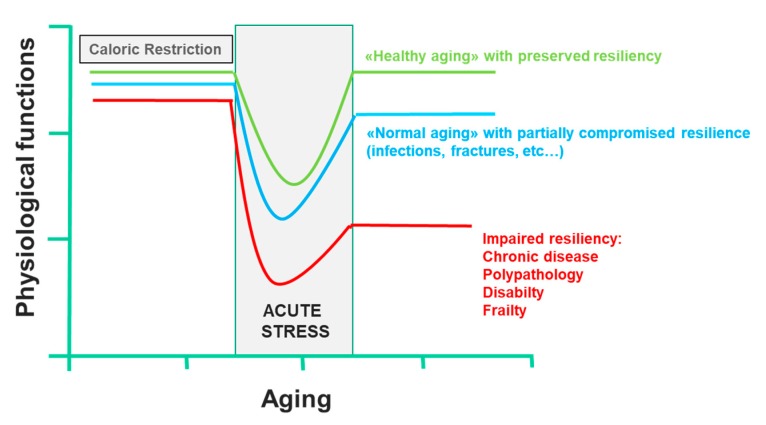
Effect of caloric restriction on aging and on the development of frailty (line 433).

Two key points are known: firstly, that most of the factors that determine lifespan are purely environmental, including diet; secondly, a CR that guarantees an adequate supply of all nutrients can prolong the life span of rodents; this effect was also demonstrated in other organisms, such as yeast, worms, flies, fish and spiders [110]. This CR paradigm has been very useful to clarify the molecular pathways that modulate aging, including the insulin-like growth factor-1 and insulin signaling pathway, the sirtuin pathway, the AMP-activated protein kinase pathway and the mammalian target of rapamycin pathway, all of which interact [52,111]. Currently, there are many substantial proofs that the combinations of CR and exercise protect against multiple molecular and cellular damages, which are the basis of the functional decline associated with aging. On the contrary, overeating and a sedentary lifestyle accelerate the functional decline associated with aging, increasing the risk of diseases. CR and physical activity increase the body’s resistance to environmental stressors and allow it to maintain its physiological function. However, in conditions of low resilience, chronic diseases, disability and frailty certainly compromise health, and consequently, the life span. In all these cases, CR not only has no beneficial effects, it also reduces the immune defenses, making response to infection inefficient and delaying the healing of wounds or fractures, reducing the resilience dramatically. Only short-term refeeding can reverse the detrimental effects of CR [112] (Figure 4). Trichopoulou et al. [84] have first proven substantially that a dietary pattern based on the MD has positive effects on life span in the elderly. They showed that a greater adherence to the MD leads to a significant reduction in mortality. More precisely, the authors showed that a greater adherence to the MD, as assessed by a semiquantitative questionnaire on food intake, was associated with a risk of death of 17% for an increase in one unit and over 50% for an increase of four units. Further evidence confirmed the association between lifestyle in middle age with successful aging and prevention against the onset of disability, frailty, and other non-communicable diseases, including cancer, and dementia [113]. It is, of course, the long-term effect of beneficial behavioral factors in middle-aged adults that contribute to successful aging, where, by successful aging, we mean the absence of important chronic diseases, severe disabilities and the preservation of cognitive functions, through the primary prevention of the onset of frailty. In contrast, a “Western” food model, which is characterized by a high intake of fried and sweet foods, processed foods and red meats, refined grains and fat-rich dairy products, is associated with a high prevalence of diseases and aging-related disabilities [7,8,9,114]. The EPIC study [115] examined the association between adherence to the MD pattern and life expectancy among 74,607 old men and women from nine European countries. The authors used a modified version of the MDS to evaluate adherence to the MD model: to calculate the monounsaturated/saturated lipid ratio, in the numerator, instead of monounsaturated lipids, the sum of monounsaturated and polyunsaturated lipids was used. In this modified MDS, both monounsaturated and polyunsaturated fatty acids are included in the numerator of the lipid ratio. The score range extends from zero (minimal adherence) to nine (maximal adherence). This change allowed the MDS score to be applied to the populations of areas other than the Mediterranean Sea basin, where notably, intake of monounsaturated fatty acids from olive oil is very low. The authors showed that for an increase of two units in the modified MDS, a reduction of 8% (95% CI: 3% to 12%) was observed for all-cause mortality. This association was naturally more evident in Greece and Spain, where the modified MDS corresponded to the traditional MD, which is followed uniformly by the whole population. However, the application of the modified MDS allowed the authors to correct any heterogeneity between countries in the association with the overall mortality scores. In Italy, most death cases occurred in northern Italy, where the traditional diet cannot be considered as an MD.

Few studies have examined the association between lifestyle and mortality among the elderly in developing countries. Shi et al. [116] analyzed data from the Chinese Longitudinal Healthy Longevity Survey (CLHLS), which involved 8959 participants aged 80 or over, in order to evaluate an association between eating habits, lifestyle factors and mortality for all causes. The authors showed that everyday fruit and vegetable consumption was significantly associated with a lower risk for global mortality (HR = 0.85; 95% CI: 0.77–0.92; *p* < 0.01; HR = 0.74; 95% CI: 0.66–0.83; *p* < 0.01, respectively). Conversely, intake of salt-preserved vegetables was associated with a higher risk of all-cause mortality (HR = 1.10; 95% CI: 1.03–1.18; *p* < 0.001). Based on these results, the authors developed a “healthy lifestyle score” based on three factors: daily intake of fruit, vegetables and regular physical activity. For each positive response to each of the three lifestyle factors, a score of 1 was assigned, for a total maximum score of 3. The subjects who led a healthier lifestyle, who are the subjects who totaled three points, had a median survival of another two years, even in the presence of chronic diseases. All the studies examined above are summarized in Table 3.

It has been confirmed by substantial evidence that MD reduces mortality from many chronic diseases, such as cardiovascular diseases, neurodegenerative diseases and even cancer [117]. All this is due to the synergistic action of the different nutritional elements of the MD. Several papers studied the functions of the nutritive components of MD in preventing cancer, obesity, cardiovascular and neurodegenerative diseases. Serra-Majem and Estruch [118] further confirmed these favorable effects of the MD in a systematic review. They confirmed the favorable effects of MD in reducing cholesterol and triglyceride levels, improving insulin resistance and increasing antioxidant capacity. In addition, the authors confirmed the favorable effects of MD on endothelium vasodilatation, metabolic syndrome, and on reducing myocardial and cardiovascular mortality and cancer incidence both in obese patients and in those with previous myocardial infarction. Rees and colleagues [119], in their systematic review of 11 randomized clinical trials in 52,044 subjects, further confirmed that the MD reduces cardiovascular disease by reducing cholesterol levels, especially LDL levels. In a further review, Sleiman et al. [120] confirmed that MD has favorable effects in reducing cardiovascular disease by reducing both fasting blood sugar and HbA1c.

Bonaccio et al., analyzing data from the Moli-sani study [121], which involved 24,325 subjects from the Molise Region, in Southern Italy, further showed that an MD pattern, that is the consumption of healthy foods with a high content of antioxidants, vitamins and phytochemicals, was significantly associated with a reduction of the 10-year cardiovascular risk. This favorable result was related to lowering levels and of glucose, lipids, CRP plasma levels, and blood pressure.

From a strictly biological point of view, there is a lot of evidence that show how nutrition works by modulating numerous interconnected cellular processes, implicated both in carcinogenesis and in inflammatory responses, or in the production of free radicals, or in the expression of inflammatory cytokines and the eicosanoid pathway [122], for example through the down-regulation of gene expression of the NF-κB transcription factor. The MD can also have a positive impact on the so-called “inflammaging” through the epigenetic mechanism (which includes chromatin remodeling, DNA methylation and miRNAs) or through the preservation of intestinal microbiota homeostasis [117].

Regarding the relationship between eating habits and mental health [123], it has been established that a diet with a high intake of vegetables, fruit, legumes as the main source of protein, olive oil as the main source of lipids, fish as the main source of animal protein, grains, nuts and seeds provides a wide range of nutrients, including vitamin B, omega-3 fatty acids and antioxidants [124]. The antioxidants contained in foods can protect brain cells from oxidative membrane damage, which has been involved in the pathogenesis of several psychiatric disorders, including depression [125], and in the pathogenesis of dementia. Regarding omega-3s, docosahexaenoic acid is essential for brain development and is highly concentrated in the brain structure. Omega-3s and vitamins are involved in a variety of brain functions, such as neurotransmitter synthesis, the development and maintenance of neuronal cells, and mechanisms to protect the blood–brain barrier [124].

## 14. Diet Patterns and “Inflammaging”

“Inflammaging” has been defined as the state of chronic activation of a low-grade inflammatory state in the elderly [126]. It has been hypothesized that it could be prodromal at the beginning of cognitive decline [127] and polypathology [128]. Most chronic diseases with a high prevalence in the elderly can most likely be correlated with an alteration of the immune response and inflammation [129]. The long-term pro-inflammatory chronic condition adversely affects survival, helping to determine a significant decline in physical performance, as well as the onset of cognitive symptoms and depression, up to defining a condition of high risk of mortality [130,131]. The role of nutrition in these processes is of great importance. While acute inflammation can contribute to increasing energy needs, comorbidities and consequent chronic low-grade inflammation are key determinants of loss of appetite and reduced nutrient intake, also known as “anorexia of aging”, thus leading to the onset of “malnutrition—related disease” [132]. The altered metabolic balance caused by malnutrition and nutrient reduction has been associated with the onset of loss in mass and muscle strength, frailty and functional dependency, resulting in disability. This creates a vicious circle between the reduced nutritional intake condition and the resulting state of a greater energy demand, determining an unfavorable prognostic pathway [133]. This condition of hyper-catabolism becomes more evident in the presence of a pathological situation in a critical state, characterized by a poor response to nutritional intervention [134]. In elderly people hospitalized for acute illness or chronic disease reactivation, the degree of inflammation, more than nutritional status, is decisive on prognosis [135]. More importantly, the low-intensity chronic hyper-catabolism status present outside the acute phase is closely related to inflammation. This condition, known as “anabolic resistance”, leads to non-optimal protein synthesis in skeletal muscle in response to physiological stimuli and is one of the main causative factors of sarcopenia [136].

## 15. Mediterranean Diet Confers Protection Against Sarcopenia

An extensive amount of evidence showed that a Mediterranean Diet pattern, or a diet with a predominant intake of vegetables, fruits, vegetable protein from legumes, and omega-3 fatty acids, especially animal protein from fish, could reduce the risk of experiencing osteoporosis and sarcopenia in the elderly. The results from a cross-sectional study involving 2570 women aged 18 to 79 years from the United Kingdom [137], have shown that a high adherence to the Mediterranean diet was significantly related to increased muscle mass and legs explosive power (LEP, watts/kg), with a significant difference of 1.7% for FFM% (fat-free mass/weight × 100) and 9.6% for LEP (trend *p* < 0.001).

A cross-sectional study conducted on 327 subjects aged over 65 from Taipei in Taiwan [138] showed that a high daily intake of mainly plant-based proteins is protective against the loss of muscle mass. As for total protein intake, subjects in the lower quartile had a greater risk of losing muscle mass than those in the upper quartile (OR = 3.03; 95% CI: 1.37–6.72). Concerning vegetable proteins intake, subjects in the lower quartile, similarly, had a higher risk of loss of muscle mass than subjects in the higher quartile (OR = 2.34, 95% CI: 1.14–4.83). With the aim to study the presence of relationships between nutrient and micronutrient intake and quality of life among sarcopenic and non-sarcopenic elderly people, the Maastricht Sarcopenia (MaSS) study [139] (227 subjects) and the PROVIDE study [140] (136 subjects) were independently conducted on two Dutch populations. Both studies showed that sarcopenics differed in certain nutritional assumptions and biochemical nutrient status compared to non-sarcopenic subjects. As regard the MaSS Study, sarcopenic subjects had a low intake of protein (*p* = 0.048), n-3 fatty acids (*p* = 0.022), folic acid (*p* = 0.016) and magnesium (*p* = 0.024), respectively, and a high intake of ALA (*p* = 0.018) (both dietary and supplement intakes were included in the study). In addition, the sarcopenic subjects had a low intake of vitamin B6 from the diet (*p* = 0.005); no significant differences were observed among sarcopenics and non-sarcopenics after including dietary supplements in the analysis (*p* = 0679). Regarding the PROVIDE study, all the sarcopenic subjects did not practice physical activity (*p* < 0.001) and had a lower quality of life compared to non-sarcopenic subjects (*p* < 0.001). Compared to the non-sarcopenic group, the sarcopenic group had a lower intake of proteins (*p* = 0.044), vitamin D (*p* = 0.007), vitamin B-12 (*p* = 0.011), magnesium (*p* = 0.015), phosphorus (*p* = 0.014) and selenium (*p* = 0.039). Lastly, Barrea et al. [141], in their cross-sectional study conducted in a sample of community-dwelling elderly women, evidenced a positive association between the adherence to the MD and muscle functional capacity measured by the Hand Grip Strength (HGS), where cut-point used for low HGS in women was <20 kg. In particular, authors showed that women with HGS < 20 Kg had a significantly lower percentage of energy intake from protein (*p* < 0.001), from carbohydrate (*p* < 0.001), and from unsaturated fat (*p* = 0.018) and n-3 PUFA (*p* = 0.031), and a significantly higher total fat (*p* < 0.001) and cholesterol intake (*p* = 0.006) than women with HGS > cut-point. They reported a strong correlation between high adherence to MD and high muscle capacity (*p* = 0.003).

All the evidence concerning the association between diet patterns and muscle mass and muscle strength is summarized in Table 4.

It must, however, be pointed out that the above studies relate the high intake of proteins, especially of vegetable origin, with a reduced risk of reduction in mass and muscle strength; the studies that considered frankly sarcopenic patients showed that this category of subjects presented a reduced protein intake, omega-3 fatty acids, and micronutrients, and a high ALA intake. It would therefore be an exaggeration to state that the Mediterranean diet certainly reduces the risk of developing sarcopenia.

In this regard, a review by Granic et al. [142] reiterated the beneficial effects of the Mediterranean diet towards the reduction of strength and muscle mass and towards the reduction of walking speed. Nevertheless, the authors suggest the need to harmonize methods for defining dietary models (i.e., Mediterranean diet vs. Healthy Eating Index), through cross-validation, together with the need for specially designed studies on different elderly populations with a longer follow-up to reach a higher level of evidence.

## 16. Mediterranean Diet Maintains Health Status and Prevents from the Onset of Frailty

Plenty of studies showed a fact already known, namely that a decisive factor regarding the development of frailty is represented by eating habits. It is known that protein supplementation, combined with physical activity with resistance exercises, are an effective way to counteract muscle weakness and physical frailty in elderly people [143].

Most authors agree that the Mediterranean diet is the best diet model that we can propose to maintain health, or to get old with a lower incidence of frailty syndrome, or disability because of chronic diseases and physical and cognitive impairment in old age. This is due to the daily intake of the main components of the MD [110,144,145].

The results of the InCHIANTI study showed that high adherence to MD was associated with better mobility performance. [146]. That is, subjects with a higher adherence to MD experienced a lower decrease in motor performance, therefore, a lower risk of developing disabilities at 3, 6 and 9 years of follow-up. Furthermore, in a subsequent study, older subjects with a high MD adherence presented a lower risk of developing frailty (OR = 0.26; 95% CI: 0.07–0.98) [147]. Regarding the protective effects of n-3 PUFA, it is known that a diet rich in n-6 PUFA, which is a precursor of arachidonic acid, which is a substrate of the enzyme cyclo-oxygenase and lipo-oxygenase, and poor in n-3 PUFA (EPA and DHA), determines a pro-inflammatory state harmful to muscles or other tissues. The integration of fish oil into a group of Brazilian women involved in a randomized trial [148] produced a better performance in lower limb strength training exercises. In the same group which was trained with supplementation of fish oil intake in the diet, a greater improvement for chair-rising performances was observed. In a subsequent study, fish oil supplementation improved walking speed in a group of postmenopausal women [149]. Another study conducted on 417 old Japanese men [150] showed that a reduced dietary EPA or DHA intake was associated with reduced motor performance. The authors therefore suggested that fish oil supplementation has a particularly beneficial effect in training against resistance and may represent a therapeutic strategy in the prevention of sarcopenia in frail patients.

Reduced synthesis of muscle proteins in older sarcopenic derives from various factors such as reduced insulin or amino acid response [151,152]. Therefore, dietary interventions must be developed with the aim to compensate the altered synthesis of muscle proteins and to help to counteract muscular atrophy caused by periods of immobilization that are frequently observed in the elderly [153]. For example, in everyday life, the distribution of protein intake is very heterogeneous between different meals: usually, less than 10 g of protein is taken during breakfast, especially among frail elderly people. [154]. This implies that for about 18 h, or the period between dinner and lunch, a frail elderly person is not getting enough protein. This explains why the anabolic response is compromised even if the daily protein intake is apparently adequate in people at risk. Results from a longitudinal study on a cohort of 690 non-institutionalized old people, from the InCHIANTI Study [155], confirmed that subjects following the traditional MD developed a significantly lower risk of frailty (OR = 0.30; 95% CI: 0.14–0.66). Authors also confirmed that a high adherence to an MD pattern was associated with a lower risk of low physical activity (OR = 0.62; 95% CI: 0.40–0.96) and low walking speed (OR = 0.48; 95% CI: 0.27–0.86). Furthermore, the results of the Seniors-ENRICA study [156] showed that high adherence to a “Prudent Pattern” diet model, that is characterized by a high intake of olive oil, vegetables, legumes, blue fish, and pasta, was related to a reduced risk of developing frailty (OR = 0.40; 95% CI: 0.20–0.81; P-trend = 0.009). On the other hand, the high level of adherence to a “Westernized Pattern” diet, that is, with a high intake of very refined flour bread, fat-rich dairy products, red or processed meat, and a poor assumption of whole-grain products, of fruit, low-fat dairy products and vegetables, correlated with a high risk of frailty (OR = 1.61; 95% CI: 0.85–3.03; P-trend = 0.14). The authors also showed that a high adherence to the “Western Pattern” diet was associated with two of Fried’s frailty criteria [13], namely reduction in walking speed (OR = 1.85; 95% CI: 1.19–2.87; P-trend = 0.007) and involuntary weight loss (OR = 2.12; 95% CI: 1.22–3.70; P-trend = 0.007).

Another Chinese study conducted on a cohort of 2724 people, men and women resident in the community, over the age of 65, examined the relationship between eating habits and the incidence of frailty [157]. The semiquantitative FFQ-questionnaire was used to evaluate dietary intake [158,159]. The authors used the Dietetic-International Index (DQI-I) scale to evaluate the quality of the diet; in a range between 0 and 94, a high score indicates a better quality of the diet [160,161]. To assess adherence to the MD, the Mediterranean Diet Score (MDS) [162] was used. After adjustment for sex and age, the authors found a 41% reduction in the risk of frailty for every increase of 10 DQI-I units (OR = 0.59; 95% CI: 0.42–0.85; *p* = 0.004). The model was also adjusted by Body Mass Index (BMI), energy intake, physical activity, education level, smoking status, alcohol use, depression, cognitive impairment, living alone and marital status at baseline. After this adjustment, the association was lower, if we consider not only the OR value, but also the *p*-value (OR = 0.69; 95% CI: 0.47–1.02; *p* = 0.056). It is interesting to note that the authors did not observe any association of MDS with frailty, among elderly Chinese people.

Two following studies showed the protective role of the MD from the development of frailty. The first study [163] was conducted on a French population from the Bordeaux cohort study of Three-City Study [164]. The semi-quantitative Food Frequency Questionnaire (FFQ) was used to evaluated food habits, while the MDS was used to evaluate the adherence to the MD [84]. A high adherence to the MD was associated, even in this study, with a reduced risk of developing frailty (*p* = 0.02). This significant decreased risk of developing frailty was also confirmed after adjusting by age, sex, marital status, education, BMI, diabetes, hypertension and cardiovascular disease history, polypharmacotherapy, cognitive functions by MMSE, and depression state. (OR = 0.32; 95% CI: 0.14–0.72, *p* = 0.006). The authors also demonstrated the association between high adherence to MD and a lower risk of frailty, considering three of Fried’s criteria [13], namely poor muscle strength (OR = 0.44; 95% CI: 0.20–0.98, *p* = 0.04), slowness (OR = 0.45; 95% CI: 0.20–50 0.99, *p* = 0.04) and low physical activity (OR = 0, 39, 95% CI: 0.18–0.82; *p* = 0.01). The second study [165] involved an American population from the Osteoarthritis Initiative (OAI) [166,167], to investigate the association between the adherence to a MD pattern and the incidence of frailty. The Block Brief 2000 food frequency (FFQ) questionnaire [168] was used to analyze participants’ diet patterns during the baseline appointment of the OAI visit. The Mediterranean diet score (aMED), validated by Panagiotakos et al. [169], was used to assess the adherence of participants to an MD model, based on the results of the FFQ. After adjusting by age, sex, race, body mass index, education, smoking habits, yearly income, physical activity level, co-morbidity and daily energy intake, authors showed that a high adherence to an MD pattern was significantly associated with a low risk of developing frailty (HR = 0.71; 95% CI: 0.50–0.99, *p* = 0.047). It is interesting to observe the association, by the authors, of a low consumption of poultry with a greater risk of frailty (HR = 1.34; 95% CI: 1.07–1.67, *p* = 0.009). All the evidence examined above are summarized in Table 5.

## 17. Conclusion Remarks on Nutrition and Frailty

Aging is the result of an interaction between local and systemic environmental factors and involutional factors due to cell senescence. A healthy lifestyle in middle age, which includes a correct diet or even a slight reduction in caloric intake, predisposes to a low risk or absence of serious chronic diseases or to the decline of cognitive or physical functions in old age. This is the consequence of preserving the efficiency of the immune system, as well as, at the cellular level, the preservation of mitochondrial activity and the reduction of oxidative stress.

**Figure 5:**
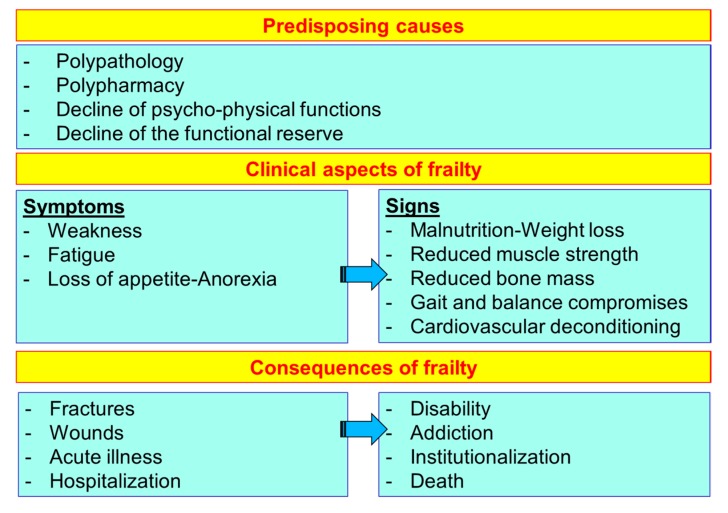
The course and consequences of frailty (line 686).

The frailty of the elderly is a condition characterized by an increased vulnerability to poor homeostasis resolution after a stress event, which increases the risk of negative outcomes, including falls, delirium and disability. Frailty must not be understood as an inevitable consequence of aging, but rather must be understood as a geriatric syndrome, or a “frailty syndrome”, that results from the interaction of various factors, including aging-related physiological alterations, poly-pathology, malnutrition, and the negative impact of socio-environmental factors. The clinical course of frailty makes its association with the state of nutrition more evident (Figure 5). In physiological aging, it is usual to observe a decrease in lean mass and bone mineral density, while a smaller reduction in fat mass was observed.

Sarcopenia is the pathological aspect of reduction of strength and muscle mass, more evident in subjects of advanced or very advanced age; this is responsible for weakness and decreased physical activity. An inadequate protein intake, which is very common among the elderly, accelerates the progression of sarcopenia. [170]. Older people very often reduce the consumption of animal proteins due to difficulty in chewing due to edentulism and the reduction of the perception of taste and smell due to polypharmacy. The expression “anorexia of aging” refers to the significant reduction in energy intake, consequent to the loss of appetite, in elderly subjects, and with a further significant reduction in protein intake. Several other factors, such as insulin resistance or altered protein digestion and amino acid absorption, inhibit the stimulation of muscle protein synthesis in the elderly, even when the dietary protein intake is adequate [171].

Reducing food intake also leads to a lack of micronutrients, contributing to the development of frailty. Very frequently, low calcium and vitamin D values are observed in the elderly population, because of insufficient dietary intake and poor sun exposure. Vitamin D and calcium deficiency accelerate bone resorption. Very often, these are subjects that expose themselves little to sunlight, thus reducing the possibility of producing vitamin D in the skin; otherwise, they show very reduced physical activity, reducing the retention of bone calcium, stimulated mainly by physical exercise. This type of behavior is further aggravated by loneliness and isolation of the elderly, a consequence of depression or, in general, by the loss of health status.

Antioxidant properties of some foods play an important role in protecting or developing frailty. Even in healthy and dwelling living elderly, a slow and gradual reduction of antioxidant defenses has been observed [172], along with an increase in oxidative stress markers [173]. This condition was certainly due to the aging process itself but it could also be the result of reduced antioxidants dietary intake [173,174]. The physiological increase of oxidative stress is certainly a responsible component of the aging process; the pathological increase of oxidative stress, resulting in reduced defense processes, is an important causal factor in the development of specific conditions of frailty, including mainly the reduction of mass and bone strength [175] and the reduction of mass and strength muscle [176]. In conclusion, the cross-sectional and prospective studies described above provide further and convincing evidence about the connection between MD adherence, or between nutrient intake with antioxidant properties and the prevention of frailty. They also contribute to increase our understanding of the molecular mechanisms involved in the physiological aging process, or in the mechanisms of protection against frailty.

## Figures and Tables

**Table 1:** Mediterranean Diet adherence and risk of mortality.

Author and Year of Publication	Study Design	Sample Size	Risk of Mortality
Trichopoulou, 2003, [84]	Population-based, prospective study	8895 men and 13,148 women	Death from any cause: HR = 0.75 (95% CI: 0.64–0.87) for a Two-Point Increase in the Mediterranean-Diet Score Death from coronary heart disease: HR = 0.67 (95% CI: 0.47–0.94) for a Two-Point Increase in the Mediterranean-Diet Score Death from cancer: HR = 0.76 (95% CI: 0.59–0.98) for a Two-Point Increase in the Mediterranean-Diet Score
Estruch, 2013, [86]	Parallel-group, multicentre, randomized trial	1050 men and 1493 women with MD with EVOO 1128 men and 1326 women with MD with nuts 987 men and 1463 women with Control Diet	Myocardial infarction, stroke, and death from cardiovascular causes: HR = 0.70 (95% CI: 0.54–0.92, *p* = 0.01) for MD with EVOO vs. Control Diet HR = 0.72 (95% CI: 0.54–0.96, *p* = 0.03) for MD with Nuts vs. Control Diet Death from any cause: HR = 0.82 (95% CI: 0.64–1.07, *p* = 0.15) for MD with EVOO vs. Control Diet HR = 0.97 (95% CI: 0.74–1.26, *p* = 0.82) for MD with Nuts vs. Control Diet
Estruch, 2018, [87]	Parallel-group, multicentre, randomized trial	1050 men and 1493 women with MD with EVOO 1128 men and 1326 women with MD with nuts 987 men and 1463 women with Control Diet	Myocardial infarction: HR = 0.82 (95% CI: 0.52–1.30) for MD with EVOO vs. Control Diet HR = 0.76 (95% CI: 0.47–1.25) for MD with Nuts vs. Control Diet Stroke: HR = 0.65 (95% CI: 0.44–0.95) for MD with EVOO vs. Control Diet HR = 0.54 (95% CI: 0.35–0.82) for MD with Nuts vs. Control Diet Death from cardiovascular causes: HR = 0.62 (95% CI: 0.36–1.06) for MD with EVOO vs. Control Diet HR = 1.02 (95% CI: 0.63–1.67) for MD with Nuts vs. Control Diet Death from any cause: HR = 0.90 (95% CI: 0.69–1.18) for MD with EVOO vs. Control Diet HR = 1.12 (95% CI: 0.86–1.47) for MD with Nuts vs. Control Diet
Sofi, 2008, [88]	Meta-analysis of prospective cohort studies	1,574,299 subjects from 12 studies	Mortality from cardiovascular diseases: RR = 0.91 (95% CI: 0.87–0.95) Mortality from any cause: RR = 0.91 (95% CI: 0.89–0.94 Mortality from cancer: RR = 0.94 (95% CI: 0.92–0.96) Incidence of Parkinson’s disease and Alzheimer’s disease: RR = 0.87 (95% CI: 0.80–0.96)
Sofi, 2010, [89]	Meta-analysis of prospective cohort studies	508,393 subjects from 7 studies	Mortality from cardiovascular diseases: RR = 0.90 (95% CI: 0.87–0.93) Mortality from any cause: RR = 0.92 (95% CI: 0.90–0.94) Mortality from cancer: RR = 0.94 (95% CI: 0.92–0.96) Incidence of neurodegenerative disease: RR = 0.87 (95% CI: 0.81–0.94)
Kromhout, 2018, [92]	Prospective Cohort Study	12,763 subjects from 16 cohorts of the Seven Countries Study	Mortality from cardiovascular diseases: Inverse association between consumption of cereals, vegetables, legumes, and alcohol and long-term CHD mortality rates (r = −0.52 to −0.62) Positive association between consumption of hard fat plus sweet products, animal foods except fish, and long-term CHD mortality rates (r = 0.68 to 0.84)

**Table 2:** Poly-unsaturated fatty acids intake and mortality.

Author and Year of Publication	Study Design	Sample Size	Risk of Mortality
GISSI Prevention trial, 1999, [93]	Prospective Cohort Study	8496 cases and 2828 controls from a cohort of 11,324 subjects	Death, non-fatal MI, and non-fatal stroke in two-way analysis: RR = 0.90 (95% CI: 0.82–0.99, *p* = 0.048) Cardiovascular death, non-fatal MI, and non-fatal stroke in two-way analysis: RR = 0.89 (95% CI: 0.80–1.01, *p* = 0.053) Death, non-fatal MI, and non-fatal stroke in four-way analysis: RR = 0.85 (95% CI: 0.74–0.98, *p* = 0.023) Cardiovascular death, non-fatal MI, and non-fatal stroke in four-way analysis: RR = 0.80 (95% CI: 0.68–0.95, *p* = 0.008)
Yokoyama, 2007, [94]	Prospective Randomised Open-Label Cohort Study	9326 EPA treatments and 9319 controls from a cohort of 18,645 subjects	Incidence of coronary events in the total study population: HR = 0.81 (95% CI: 0.69–0.95, *p* = 0.011) for EPA treatments vs. controls; Incidence of coronary events in in the primary prevention arm: HR = 0.82 (95% CI: 0.63–1.06, *p* = 0.132) for EPA treatments vs. controls; Incidence of coronary events in in the secondary prevention arm: HR = 0.81 (95% CI: 0.66–1.00, *p* = 0.048) for EPA treatments vs. controls
Kromhout, 2010, [95]	Prospective Multi-centre, double-blind trial: n−3 fatty acids EPA and DHA and plant-derived ALA vs. placebo	1212 subjects randomized to receive EPA–DHA and ALA; 1192 subjects randomized to receive EPA–DHA and ALA placebo; 1197 subjects randomized to receive EPA–DHA placebo and ALA; 1236 subjects randomized to receive EPA–DHA placebo and ALA placebo	Major cardiovascular events: HR = 1.01 (95% CI: 0.87–1.17, *p* = 0.93) with EPA–DHA; HR = 0.91 (95% CI: 0.78–1.05, *p* = 0.20) with ALA
Einvik, 2010, [96]	Interventional Clinical Trial	563 Norwegian men randomized to a 3-year clinical trial of diet with n-3 PUFA supplementation vs. placebo (corn oil)	Mortality from any cause: HR = 0.57 (95% CI: 0.29–1.10) Mortality from cardiovascular diseases: HR = 0.86 (95% CI: 0.57–1.38)
Bosch, 2012, [97]	Prospective multi-centre, double-blind trial: n−3 fatty acids vs. placebo	6281 subjects randomized to receive n−3 fatty acids; 6255 subjects randomized to receive placebo	Death from cardiovascular causes: HR = 0.98 (95% CI: 0.87–1.10, *p* = 0.72) Myocardial Infarction, Stroke, or Cardiovascular Death: HR = 1.01 (95% CI: 0.93–1.10, *p* = 0.81) Death from Any Cause: HR = 0.98 (95% CI: 0.89–1.07, *p* = 0.63) Death from Arrhythmia: HR = 1.10 (95% CI: 0.93–1.30, *p* = 0.26)
Rauch, 2010, [98]	Prospective randomized, placebo-controlled, double-blind, multicentre trial	1919 subjects randomized to receive n−3 fatty acids; 1885 subjects randomized to receive placebo	Sudden cardiac death: OR = 0.95 (95% CI: 0.56–1.60, *p* = 0.84) Total mortality: OR = 1.25 (95% CI: 0.90–1.72, *p* = 0.18) Major adverse cerebrovascular and cardiovascular Events: OR = 1.21 (95% CI: 0.96–1.52, *p* = 0.10) Revascularization in survivors: OR = 0.93 (95% CI: 0.80–1.08, *p* = 0.34)
Galan, 2010, [99]	Prospective randomized, placebo-controlled, double-blind trial	620 subjects randomized to receive B vitamins + omega 3 fatty acids; 633 subjects randomized to receive Omega 3 fatty acids; 622 subjects randomized to receive B vitamins; 626 subjects randomized to receive placebo	Non-fatal myocardial infarction, stroke, or death from cardiovascular disease: HR = 1.08 (95% CI: 0.79–1.47, *p* = 0.64); Total mortality: HR = 1.03 (95% CI: 0.72–1.48, *p* = 0.88)
Bonds, 2014, [100]	2 × 2 factorial-designed randomized clinical trial	1079 subjects randomized to receive lutein + zeaxanthin and DHA + EPA; 1068 subjects randomized to receive DHA + EPA; 1044 subjects randomized to receive lutein + zeaxanthin; 1012 subjects randomized to receive placebo	Time to First Cardiovascular Disease Mortality/Morbidity Event: HR = 0.95 (95% CI: 0.78–1.17) for DHA + EPA vs. No DHA + EPA; HR = 0.94 (95% CI: 0.77–1.15) for Lutein + zeaxanthin vs. No Lutein + zeaxanthin
Deepak, 2019, [101]	Multicentre, randomized, double-blind, placebo-controlled trial	4089 subjects randomized to receive 2 g of Icosapent Ethyl twice daily; 4090 subjects randomized to receive placebo	Cardiovascular death, nonfatal myocardial infarction, nonfatal stroke, coronary revascularization, or unstable angina: HR = 0.75 (95% CI: 0.68–0.83, *p* < 0.001)
Bucher, 2002, [102]	Meta-analysis from 11 case-control studies	7951 patients in the treatment groups and 7855 patients in the control groups	Nonfatal myocardial infarction: RR = 0.80 (95% CI: 0.5–1.2, *p* = 0.16) for n-3 poly-unsaturated fatty acid-enriched diets; Fatal myocardial infarction: RR = 0.70 (95% CI: 0.6–0.8, *p* < 0.001) for n-3 poly-unsaturated fatty acid-enriched diets; Sudden death: RR = 0.70 (95% CI: 0.6–0.9, *p* < 0.01) for n-3 poly-unsaturated fatty acid-enriched diets; Overall mortality: RR = 0.80 (95% CI: 0.7–0.9, *p* < 0.001) for n-3 poly-unsaturated fatty acid-enriched diets
Rizos, 2012, [103]	Meta-analysis from 20 case-control studies	34,388 patients in the treatment groups and 34,292 patients in the control groups	All-cause mortality: RR = 0.96 (95% CI: 0.91–1.02) for n-3 poly-unsaturated fatty acids; Cardiac death: RR = 0.91 (95% CI: 0.85–0.98) for n-3 poly-unsaturated fatty acids; Sudden death: RR = 0.87 (95% CI: 0.75–1.01) for n-3 poly-unsaturated fatty acids; Myocardial infarction: RR = 0.89 (95% CI: 0.76–1.04) for n-3 poly-unsaturated fatty acids; Stroke: RR = 1.05 (95% CI: 0.93–1.18) for n-3 poly-unsaturated fatty acids
Kwak, 2012, [104]	Meta-analysis from 14 placebo-control trials	10,226 patients in the treatment groups and 10,259 patients in the control groups	Overall cardiovascular events: RR = 0.99 (95% CI: 0.89–1.09) for omega-3 fatty acid supplement; All-cause mortality: RR = 0.96 (95% CI: 0.90–1.02) for omega-3 fatty acid supplement; Sudden cardiac death: RR = 0.93 (95% CI: 0.66–1.30) for omega-3 fatty acid supplement; Cardiovascular death: RR = 0.92 (95% CI: 0.35–1.01) for omega-3 fatty acid supplement; Myocardial infarction: RR = 0.81 (95% CI: 0.65–1.01) for omega-3 fatty acid supplement; Angina and unstable angina: RR = 0.77 (95% CI: 0.50–1.18) for omega-3 fatty acid supplement; Congestive heart failure: RR = 0.92 (95% CI: 0.73–1.17) for omega-3 fatty acid supplement; Transient ischemic attack and Stroke: RR = 1.13 (95% CI: 0.77–1.66) for omega-3 fatty acid supplement
Agency for Healthcare Research and Quality, 2016, [105]	Meta-analysis from 61 randomized controlled trials and 37 longitudinal observational studies	No available data about sample sizes of cohorts examined	All-cause death: HR = 0.97 (95% CI: 0.92–1.03) for EPA + DHA; Major Adverse Cardiovascular Events: HR = 0.96 (95% CI: 0.91–1.02) for EPA + DHA; Myocardial infarction: HR = 0.88 (95% CI: 0.77–1.02) for EPA + DHA; Cardiovascular Disease Death: HR = 0.92 (95% CI: 0.82–1.02) for EPA + DHA; Sudden Cardiac Death: HR = 1.04 (95% CI: 0.92–1.17) for EPA + DHA; Stroke: HR = 0.98 (95% CI: 0.88–1.09) for EPA + DHA
Zhang, 2018, [106]	Prospective cohort study	Total and cause-specific Mortality from a cohort of 240,729 men and 180,580 women	All-cause death: HR = 0.89 (95% CI: 0.86–0.92, *p* < 0.0001) for highest vs. lowest quintiles of long-chain omega-3 PUFAs intake in men; HR = 0.90 (95% CI: 0.86–0.94, *p* < 0.0001) for highest vs. lowest quintiles of long-chain omega-3 PUFAs intake in women; Cancer death: HR = 0.95 (95% CI: 0.90–1.00, *p* = 0.040) for highest vs. lowest quintiles of long-chain omega-3 PUFAs intake in men; HR = 1.01 (95% CI: 0.93–1.09, *p* = 0.51) for highest vs. lowest quintiles of long-chain omega-3 PUFAs intake in women; Cardiovascular disease death: HR = 0.85 (95% CI: 0.80–0.90, *p* < 0.0001) for highest vs. lowest quintiles of of long-chain omega-3 PUFAs intake in men; HR = 0.82 (95% CI: 0.75–0.90, *p* < 0.0001) for highest vs. lowest quintiles of long-chain omega-3 PUFAs intake in women; Respiratory disease death: HR = 0.73 (95% CI: 0.65–0.83, *p* < 0.0001) for highest vs. lowest quintiles of long-chain omega-3 PUFAs intake in men; HR = 0.74 (95% CI: 0.64–0.87, *p* < 0.0001) for highest vs. lowest quintiles of long-chain omega-3 PUFAs intake in women; Alzheimer’s Disease death: HR = 0.70 (95% CI: 0.54–0.89, *p* = 0.0008) for highest vs. lowest quintiles of long-chain omega-3 PUFAs intake in men; HR = 0.59 (95% CI: 0.43–0.80, *p* = 0.0024) for highest vs. lowest quintiles of long-chain omega-3 PUFAs intake in women; Chronic liver disease death: HR = 0.66 (95% CI: 0.49–0.89, *p* = 0.0046) for highest vs. lowest quintiles of long-chain omega-3 PUFAs intake in men; HR = 1.30 (95% CI: 0.78–2.16, *p* = 0.88) for highest vs. lowest quintiles of long-chain omega-3 PUFAs intake in women

**Table 3:** Mediterranean diet components and healthy aging.

Author and Year of Publication	Study Design	Sample Size	Risk of Mortality
Trichopoulou, 1995, [85]	Prospective cohort study	91 men and 91women	Mortality Rate: RR = 0.83 (95% CI: 0.69–0.99, *p* = 0.04) for high adherence to MD
Britton, 2008, [7]	Longitudinal cohort study	4140 men and 1823 women	Likelihood of Successful Aging for men: OR = 1.52 (95% CI: 1.34–1.72, *p* < 0.001) for socioeconomic position OR = 1.19 (95% CI: 1.06–1.33, *p* = 0.003) for early-life factors OR = 1.29 (95% CI: 1.16–1.44, *p* < 0.001) for health behaviours OR = 1.12 (95% CI: 1.01–1.24, *p* = 0.03) for psychosocial factors Likelihood of Successful Aging for women: OR = 1.58 (95% CI: 1.31–1.92, *p* < 0.001) for socioeconomic position. OR = 1.23 (95% CI: 1.01–1.49, *p* = 0.04) for early-life factors OR = 1.29 (95% CI: 1.09–1.54, *p* = 0.003) for health behaviours OR = 1.10 (95% CI: 0.94–1.28, *p* = 0.25) for psychosocial factors
Akbaraly, 2013, [8]	Longitudinal cohort study	3775 men and 1575 women	Ideal Aging with Healthy-foods diet: OR = 1.19 (95% CI: 0.82–1.73, *p* = 0.35) for higher vs. lower tertile; Non-fatal cardiovascular disease with Healthy-foods diet: OR = 1.10 (95% CI: 0.89–1.35, *p* = 0.39) for higher vs. lower tertile; Cardiovascular disease death with Healthy-foods diet: OR = 0.66 (95% CI: 0.43–1.01, *p* = 0.05) for higher vs. lower tertile; Non-cardiovascular disease death with Healthy-foods diet: OR = 0.61 (95% CI: 0.47–0.80, *p* < 0.0001) for higher vs. lower tertile; Ideal Aging with Western-type diet: OR = 0.52 (95% CI: 0.33–0.82, *p* = 0.005) for higher vs. lower tertile; Non-fatal cardiovascular disease with Western-type diet: OR = 1.08 (95% CI: 0.83–1.41, *p* = 0.56) for higher vs. lower tertile; Cardiovascular disease death with Western-type diet: OR = 1.66 (95% CI: 0.95–2.89, *p* = 0.07) for higher vs. lower tertile; Non-cardiovascular disease death with Western-type diet: OR = 1.23 (95% CI: 0.87–1.72, *p* = 0.24) for higher vs. lower tertile
Samieri, 2013, [9]	Cross-sectional observational study	1171 “Healthy agers” vs. 9499 “Usual agers”	Healthy aging and component of healthy aging, according to Alternative Healthy Eating Index-2010: Healthy aging: OR = 1.34 (95% CI: 1.09–1.66, *p* < 0.001) for higher vs. lower quintile; No chronic disease: OR = 1.01 (95% CI: 0.97–1.05, *p* = 0.26) for higher vs. lower quintile; No cognitive impairment: OR = 0.99 (95% CI: 0.97–1.01, *p* = 0.09) for higher vs. lower quintile; No impairment of physical function: OR = 1.23 (95% CI: 1.11–1.36, *p* < 0.001) for higher vs. lower quintile; No limitation of mental health: OR = 1.13 (95% CI: 1.05–1.22, *p* < 0.001) for higher vs. lower quintile; Healthy aging and component of healthy aging, according to MD: Healthy aging: OR = 1.46 (95% CI: 1.17–1.83, *p* = 0.0022) for higher vs. lower quintile; No chronic disease: OR = 1.04 (95% CI: 1.00–1.09, *p* = 0.13) for higher vs. lower quintile; No cognitive impairment: OR = 0.97 (95% CI: 0.95–1.00, *p* = 0.02) for higher vs. lower quintile; No impairment of physical function: OR = 1.14 (95% CI: 1.03–1.26, *p* = 0.005) for higher vs. lower quintile; No limitation of mental health: OR = 1.12 (95% CI: 1.04–1.20, *p* < 0.001) for higher vs. lower quintile
Trichopoulou, 2005, [115]	Multicentre, prospective cohort study	24,545 men and 50,062 women from the EPIC-elderly cohort	Mortality ratios (MR) for all countries: MR = 0.92 (95% CI: 0.88–0.97, *p* value for heterogeneity = 0.328) for 2 unit increase of modified MD score; Mortality ratios (MR) calibrated across countries: MR = 0.93 (95% CI: 0.88–0.99, *p* value for heterogeneity = 0.091) for 2 unit increase of modified MD score
Shi, 2015, [116]	Longitudinal cohort study	3567 men and 5392 women from the Chinese Longitudinal Healthy Longevity Survey (CLHLS)	Hazard ratios for all-cause mortality: HR = 0.73 (95% CI: 0.68–0.77, *p* < 0.01) for physical activity vs. no physical activity; HR = 0.85 (95% CI: 0.77–0.92, *p* < 0.01) for daily fruit intake; HR = 0.74 (95% CI: 0.66–0.83, *p* < 0.01) for daily vegetable intake; HR = 1.05 (95% CI: 0.97–1.14, *p* > 0.05) for daily meat intake; HR = 1.06 (95% CI: 1.00–1.13, *p* < 0.05) for occasionally fish intake; HR = 1.04 (95% CI: 0.97–1.12, *p* > 0.05) for daily sugar intake; HR = 1.10 (95% CI: 1.03–1.18, *p* < 0.01) for daily salt-preserved vegetable intake

**Table 4:** Mediterranean diet pattern, muscle mass and muscle strength.

Author and Year of Publication	Study Design	Sample Size	Muscle Mass and Muscle Strength
Kelaiditi, 2016, [137]	Cross-sectional study	2570 women from the Twins UK study	Fat-free mass (%): 0.9 ± 0.4 P-trend = 0.012 for highest vs. lowest adherence to MD in women ≤ 50 years; 1.0 ± 0.4 P-trend = 0.008 for highest vs. lowest adherence to MD in women ≥ 50 years; Grip strength (kg): 0.3 ± 1.0 P-trend = 0.912 for highest vs. lowest adherence to MD in women ≤ 50 years; −0.1 ± 0.5 P-trend = 0.975 for highest vs. lowest adherence to MD in women ≥ 50 years; Leg explosive power (watts/kg): 7.4 ± 3.2 P-trend = 0.010 for highest vs. lowest adherence to MD in women ≤ 50 years; 9.5 ± 3.0 P-trend = 0.005 for highest vs. lowest adherence to MD in women ≥ 50 years
Huang, 2016, [138]	Cross-sectional study	327 community-dwelling elderly people	Odds ratios for total protein and vegetable protein density for Low Muscle Mass (LMM): OR = 3.11 (95% CI: 1.42–6.84, *p* = 0.005) for lowest vs. highest total protein density intake; OR = 2.50 (95% CI: 1.22–5.10, *p* = 0.012) for lowest vs. highest vegetable protein density intake; Adjusted least square (LS) means for LMM vs. normal groups: 14.5 vs. 15.5, *p* = 0.008 for total protein density intake; 7.0 vs. 8.2, *p* = 0.002 for vegetable protein density intake
Ter Borg, 2016, [139]	Cross-sectional study	227 community-dwelling adults aged over 65 years from the Maastricht Sarcopenia Study	Mean(SD) of daily dietary and supplement intake of nutrients for sarcopenic vs. nonsarcopenic subjects: Protein (g): 68 (22) vs. 74 (20), *p* = 0.048; N-3 fatty acids (g): 1.7 (0.7) vs. 2.1 (0.8), *p* = 0.005; ALA, 18:3n-3 (g): 1.47 (0.59) vs. 1.73 (0.72), *p* = 0.018; Folic acid equivalents (g): 312 (160) vs. 375 (167), *p* = 0.016 Magnesium (mg): 305 (132) vs. 350 (125), *p* = 0.024; Mean(SD) of biochemical nutrient levels for sarcopenic vs. nonsarcopenic subjects: 25-hydroxyvitamin D (nmol/l): 56.2 (31.3) vs. 70.1 (30.3), *p* = 0.004; EPA, 20:5n-3(%): 0.79 (0.27) vs. 0.94 (0.38), *p* = 0.007; LA, 18:2n-6, %: 10.6 (1.6) vs. 9.9 (1.6), *p* = 0.016; Homocysteine, mmol/l: 12.1 (4.2) vs. 15.2 (7.9), *p* < 0.001
Verlaan, 2017, [140]	Matched case-control observational study	66 sarcopenic older adults vs. 66 non-sarcopenic older adults from the PROVIDE Study	Mean (SD) of daily dietary nutrient intakes for sarcopenic vs. nonsarcopenic subjects: Protein (g): 72.5 (19.6) vs. 75.3 (20.7), *p* = 0.359; Protein (g/kg): 0.99 (0.24) vs. 1.0 9 (0.29), *p* = 0.044 Carbohydrate (g): 212 (61) vs. 208 (76), *p* = 0.906; Total Fat (g): 63.3 (19.0) vs. 65.8 (22.1), *p* = 0.403; Vitamin B-12 (g): 3.9 (2.6) vs. 5.3 (3.6), *p* = 0.011 Vitamin D (mg): 2.6 (2.1) vs. 4.0 (3.4), *p* = 0.007 Magnesium (mg): 260 (96) vs. 295 (86), *p* = 0.015; Phosphorus (mg): 1196 (330) vs. 1325 (338), *p* = 0.014 Selenium (mg): 39.1 (17.1) vs. 46.5 (21.2), *p* = 0.039
Barrea, 2019, [141]	Cross-sectional observational study	84 not hospitalized elderly women from the PERSSILAA project	Daily nutrients (SD, range) intake of participants according the HGS cut-point: Protein (%): 12.24 (2.04) for HGS < 20 Kg vs. 14.75 (1.45) for HGS > 20 Kg, *p* < 0.001; Carbohydrate (%): 55.1 (range 50.91–60.00) for HGS < 20 Kg vs. 56.00 (range 51.00–61.90) for HGS > 20 Kg, *p* < 0.001; Total Fat (%): 32.34 (3.38) for HGS < 20 Kg vs. 29.50 (3.27) for HGS > 20 Kg, *p* < 0.001; Unsaturated Fat (%): 20.98 (3.96) for HGS < 20 Kg vs. 22.83 (3.05) for HGS > 20 Kg, *p* = 0.018; N-3 PUFA (g/day): 4.28 (2.85) for HGS < 20 Kg vs. 5.54 (2.42) for HGS > 20 Kg, *p* = 0.031; Cholesterol (mg/day): 332.42 (34.91) for HGS < 20 Kg vs. 309.78 (38.24) for HGS > 20 Kg, *p* = 0.006; Association of adherence to MD with the HGS, after adjusting for BMI: Low adherence to MD: OR = 0.73 (95% CI: 0.61–0.86), *p* < 0.001; Average adherence to MD: OR = 1.02 (95% CI: 0.95–1.09), *p* = 0.611 High adherence to MD: OR = 1.14 (95% CI: 1.04–1.25), *p* = 0.003

**Table 5:** Mediterranean diet components and frailty.

Author and Year of Publication	Study Design	Sample Size	Risk of Frailty
Milaneschi, 2011, [146]	Prospective population-based study	935 community-living subjects aged over 65 years from the InCHIANTI Study cohort	Adjusted odds of developing mobility disability: OR = 0.73 (95% CI: 0.41–1.28, *p* = 0.27) for highest vs. lowest adherence to MD; Decrease in SPPB scores at 9 years of follow up: Average Score = 0.9 (SE = 0.41, *p* = 0.03) for highest vs. lowest adherence to MD; Adjusted incidence of mobility disability: HR = 0.71 (95% CI: 0.51–0.98, *p* = 0.04) for highest vs. lowest adherence to MD
Bollwein, 2013, [147]	Cross-sectional study	192 community-dwelling volunteers aged over 75 years	Odds Ratio for Frailty: OR = 0.19 (95% CI: 0.05–0.82, *p* = 0.011) for highest vs. lowest adherence to MD
Talegawkar, 2012, [155]	Prospective population-based study	690 community-living subjects aged over 65 years from the InCHIANTI Study cohort	Odds Ratio for Frailty: OR = 0.30 (95% CI: 0.14–0.66) for highest vs. lowest adherence to MD
Luz, 2015, [156]	Prospective cohort study	1872 non-institutionalized subjects aged over 60 years from the Seniors-ENRICA cohort Study	Odds Ratio for Frailty: OR = 0.40 (95% CI: 0.20–0.81, *p* = 0.009) for highest adherence to a “prudent pattern” diet; 0.40 (0.20–0.81) 0.009 OR = 1.61 (95% CI: 0.85–3.03, *p* = 0.14) for highest adherence to a “westernized pattern” diet
Rahi, 2017, [163]	Population-based prospective cohort study	560 non-institutionalized subjects aged over 65 years from the cohort of Three-City-Bordeaux Study	Odds Ratio for Frailty: OR = 0.32 (95% CI: 0.14–0.72, *p* = 0.006) for highest vs. lowest adherence to MD
Veronese, 2017, [165]	Population-based prospective cohort study	1857 men and 2564 women from the The Osteoarthritis Initiative cohort Study	Odds Ratio for Frailty: OR = 0.71 (95% CI: 0.50–0.99, *p* = 0.047) for highest vs. lowest adherence to MD
